# Efficacy of Transversus Abdominis Plane Block in the Reduction of Pain and Opioid Requirement in Laparoscopic and Robot-assisted Hysterectomy: A Systematic Review and Meta-analysis

**DOI:** 10.1055/s-0041-1740595

**Published:** 2022-01-29

**Authors:** Claudia López-Ruiz, Jerutsa Catalina Orjuela, Diego Fernando Rojas-Gualdrón, Marcela Jimenez-Arango, José Fernando de los Ríos, Elsa Maria Vásquez-Trespalacios, Claudia Vargas

**Affiliations:** 1 Department of Gynecology and Obstetrics, Clínica del Prado, Medellín, Colombia; 2 School of Graduate Studies, School of Medicine, CES University, Medellín, Colombia; 3 American Association of Gynecologic Laparoscopists, Medellín, Colombia

**Keywords:** transversus abdominis plane block, pain, laparoscopic hysterectomy, robotic-assisted hysterectomy, opioid, bloqueio do plano transverso do abdome, dor, histerectomia laparoscópica, histerectomia robótica assistida, opioide

## Abstract

**Objective** To summarize the available evidence of TAP Block in efficacy in laparoscopic or robotic hysterectomy.

**Data Sources** We searched databases and gray literature for randomized controlled trials in which transversus abdominis plane (TAP) block was compared with placebo or with no treatment in patients who underwent laparoscopic or robot-assisted hysterectomy.

**Method of Study Selection** Two researchers independently evaluated the eligibility of the selected articles.

**Tabulation, Integration, and Results** Seven studies were selected, involving 518 patients. Early postoperative pain showed a difference in the mean mean difference (MD): - 1.17 (95% confidence interval [CI]: - 1.87–0.46) in pain scale scores (I ^2^ = 68%), which was statistically significant in favor of using TAP block, but without clinical relevance; late postoperative pain: DM 0.001 (95%CI: - 0.43–0.44; I ^2^ = 69%); opioid requirement: DM 0.36 (95%CI: - 0.94–1.68; I ^2^ = 80%); and incidence of nausea and vomiting with a difference of 95%CI = - 0.11 (- 0.215–0.006) in favor of TAP.

**Conclusion** With moderate strength of evidence, due to the high heterogeneity and imbalance in baseline characteristics among studies, the results indicate that TAP block should not be considered as a clinically relevant analgesic technique to improve postoperative pain in laparoscopic or robotic hysterectomy, despite statistical significance in early postoperative pain scale scores.

**Clinical Trial Number and Registry:** PROSPERO ID - CRD42018103573.

## Resumo

**Objetivo** Resumir as evidências disponíveis sobre a eficácia do bloqueio TAP em histerectomia laparoscópica ou robótica.

**Fontes de Dados** Pesquisamos bancos de dados e literatura cinza por ensaios clínicos randomizados nos quais o bloqueio do plano transverso do abdome (TAP na sigla em inglês) foi comparado com placebo ou com nenhum tratamento em pacientes que foram submetidos a histerectomia laparoscópica ou assistida por robô.

**Métodos de Seleção de Estudos** Dois pesquisadores avaliaram independentemente a elegibilidade dos artigos selecionados.

**Tabulação, Integração e Resultados** Sete estudos foram selecionados envolvendo 518 pacientes. A dor pós-operatória precoce apresentou diferença nas médias (DM) de: -1 17 (intervalo de confiança [IC] de 95%: - 1 87–0 46) nos escores da escala de dor (I2 = 68%) o que foi estatisticamente significativo a favor do uso do bloqueio TAP mas sem relevância clínica; dor pós-operatória tardia: DM 0001 (IC95%: - 043–044; I2 = 69%); necessidade de opioides: DM 0 36 (95%CI: - 0 94–168; I2 = 80%); e incidência de náuseas e vômitos com diferença de 95% CI = - 011 (- 0215–0006) a favor do TAP.

**Conclusão** Com moderada força de evidência devido à alta heterogeneidade e ao desequilíbrio nas características basais entre os estudos os resultados indicam que o bloqueio do TAP não deve ser considerado como uma técnica analgésica clinicamente relevante para melhorar a dor pós-operatória em histerectomia laparoscópica ou robótica apesar da significância estatística nas pontuações da escala de dor pós-operatória inicial.

## Introduction

Hysterectomy is a surgical procedure often associated with significant postoperative pain. This could be attributed to injuries suffered in the pelvic plexus, 1 which is predominantly composed of neural structures in the sacral and lower lumbar segments, as well as to inflammation caused by direct trauma to tissues during the surgical procedure. 2 Therefore, it has been considered that the block in the transverse abdominal plane (TAP), which acts by blocking iliohypogastric, ilioinguinal, and lower thoracic spinal nerves, could be useful. However, the pain referred by patients after a hysterectomy is more of a visceral origin.

Despite laparoscopic and robotic hysterectomy being minimally invasive surgeries, only 60% of the patients feel satisfied with postoperative pain control in such gynecologic procedures. 3 The use of analgesics and of nonsteroidal anti-inflammatory drugs has been the first line recommendation for the management of postoperative pain after hysterectomy. 4 Hence, the use of analgesic medications, including opioids, which are associated with minor side effects (pruritus, nausea, and vomiting), as well as major side effects (respiratory depression and addiction), 5 are sometimes required, increasing postoperative morbidity and mortality. 6 7

To reduce postoperative pain and opioid side effects, several strategies have been developed in the context of multimodal analgesia. 8 Among them is TAP 9 block, in which local anesthetic is injected into the neurovascular plane between the internal oblique and transversus muscles of the abdominal wall, with the goal of blocking the lower thoracic spinal nerves (T7-T12) and the iliohypogastric and ilioinguinal nerves (L1). 9

Since TAP block was first described in 2001 by Rafi, 9 its efficacy has been evaluated in multiple clinical trials and compared with other analgesic techniques in patients undergoing abdominal and pelvic procedures, including hysterectomy performed through several approaches: abdominal, 10 11 12 laparoscopic, 13 14 15 16 17 18 and robot-assisted. 19 20 21

Currently, there are only two available studies evaluating the efficacy of TAP block exclusively in the context of surgical approaches to hysterectomy. In the first meta-analysis, Tubog et al. 22 reported a reduction in pain during the first postoperative hours. On the other hand, a second meta-analysis by Bacal et al. 23 showed that postoperative pain was reduced for 24 hours. Regarding opioid dosing, Tubog et al. 22 found that TAP block had opioid-sparing effects in all surgical approaches, while the study by Bacal et al. 23 reported the opioid-sparing effect of TAP block only in patients undergoing abdominal hysterectomy, but not in those undergoing laparoscopic hysterectomy. However, the results obtained presented a significant heterogeneity.

In the present systematic review and meta-analysis, we have evaluated the best available evidence of the efficacy of TAP block in reducing pain and opioid requirement exclusively in laparoscopic and robot-assisted hysterectomy, taking into account and exploring the substantial data heterogeneity identified when drawing conclusions.

## Methods

The present study was designed following the recommendations of the Preferred Reporting Items for Systematic Reviews and Meta-Analyses (PRISMA) statement. 24

### Protocol and Registration

The present review was based in a previously registered and developed review protocol, which was prepared following the Cochrane Handbook for Systematic Reviews of Interventions, 25 and may be found under PROSPERO ID: CRD42018103573.

### Eligibility Criteria

The PICO format was used to locate the evidence addressing the clinical query; (P) patients who underwent laparoscopic or robot-assisted hysterectomy for benign or malignant disease; (I) intervened with TAP block in laparoscopic or robot-assisted hysterectomy; (C) compared with placebo or with no treatment; (O): studies measuring any pain scale and opioid requirement; (S) randomized, blinded clinical trials. Studies published until to July 31 ^st^ , 2018, without language restriction, were considered eligible for the present analysis.

### Information Sources and Search

We searched the following electronic databases, trial registers, and websites: PubMed, Embase, LILACS, Cochrane Database of Systematic Reviews (CDSR), Clinical trials Web site ( www.clinicaltrials.gov ), SCIELO, Google Scholar, and Open Gray. The search strategy in PubMed included the following terms: *transversus abdominis plane* *block* OR *TAP block* AND *hysterectomy* . The same strategy was used for other databases, changing only syntaxes.

### Study Selection

After eliminating studies registered in more than one database, those considered irrelevant according to the inclusion criteria or due to repeated publication were also excluded. This was a two-step process; an initial screening of titles and abstracts, and a second screening that was performed by reading the full texts. Two of the review authors (C.C.L and Orjuela J. C.) independently performed this process, and disagreements were resolved by consensus.

### Data Collection Process and Data Items

As a primary outcome, the efficacy of TAP block in terms of postoperative pain was evaluated. Pain was assessed by the visual analogue scale (VAS), in which scores range from 0 to 10, where 0 is absence of pain and 10 is the maximum perceived pain. Data from studies reporting scores from 0 to 100 were converted to 0 to 10 by dividing the scores by 10. Pain was assessed at 2 time points: early (1 to 4 hours after surgery) and late (24 hours after surgery). As a secondary outcome, we evaluated the use of opioid during the first 24 postoperative hours and the side effects of their use were described, specifically nausea, vomiting, and sedation. Likewise, quality of recovery was also reported.

Two of the review authors (C.C.L. and Orjuela J. C.) independently extracted the previously described data into a Microsoft Excel (Microsoft Corporation, Redmond, WA, USA) spreadsheet. The extracted data included: the country where the study was performed, approach, inclusion and exclusion criteria, evaluated outcomes, blinding, sequence generation and concealment, number of patients, type of intervention, technique used to administer block, medication used, comparison group intervention, patient characteristics (age, body mass index [BMI]), American Society of Anesthesiology (ASA) score, preoperative and postoperative pain evaluated trough the visual analogue scale (VAS) scale and evaluation of pain, and reporting of side effects associated with opioids (nausea, vomiting, drowsiness), as well as quality of recovery. In case the required measurements were not identified, or if the results were exclusively reported as figures, the authors of the studies were contacted, and if no response was obtained, data were estimated by means of WebPlotDigitizer ( https://automeris.io/WebPlotDigitizer/ ).

### Risk of Bias in Individual Studies

Two of the review authors (C.C.L and Orjuela J. C.) independently assessed the included studies for risk of bias using the Cochrane “Risk of bias” assessment tool (RoB 2.0) 26 for the six following domains: bias arising from the randomization process, bias due to deviations from intended interventions, bias due to missing outcome data, bias in the measurement of the outcome, bias in the selection of the reported result, and other bias. Each of the two review authors classified studies as being of high, undetermined, or low risk, according to the algorithm of the tool. This classification was discussed and consensual with a third investigator (Rojas-Gualdrón D. F.).

### Summary of Measures, Synthesis of Results, and Statistical Analysis

The primary outcome was the mean difference between early (4 hours) and late (24 hours) postoperative pain scale scores. A reduction in the pain scale score of 2 to 2.7 points, or of 30 to 40%, was considered to be significant, according to meaningful pain reduction for the patients. 27 28 29 30 As secondary outcome, differences of mean total morphine use in the first 24 hours after surgery were analyzed. We did not need to apply opioid conversion, since all analyzed studies exclusively used morphine. In addition, the difference in proportion of adverse events (nausea/vomiting) was analyzed in studies reporting this data. In case a study did not report means, but reported medians instead, estimations were made following the procedure described by Wan et al. based on sample size, medians, and interquartile ranges. 31

Based on the reported outcomes in the studies classified as of low risk of bias, the weighted estimate was obtained together with 95% confidence intervals (CIs) by the weighted least squares method, which is more robust than conventional random effects in the presence of publication bias (small sample) and than fixed effects in the presence of heterogeneity. 32 Heterogeneity among studies was calculated by tau (absolute) and I ^2^ (relative).

### Risk of Bias across Studies and Sensitivity Analyses

The individual contribution of each study to the heterogeneity among studies was evaluated by means of sensitivity analysis by calculating the I ^2^ value when each individual study was excluded, and the possibility that baseline heterogeneity could explain the observed heterogeneity among studies was analyzed following the method described by Hicks et al. 33 Risk of publication bias was assessed visually by funnel plot augmentation, and no statistical test was performed given the low number of studies included in the meta-analysis. 34

Two sensitivity analyses were performed. First, the prediction interval was estimated to evaluate between study heteogenity in the mean difference scale, 34 and by augmented funnel plots, the possible scenarios of weighted outcomes when updating the meta-analysis were estimated and analyzed and were grouped according to the possible outcomes of the hypothesis with a significance level of 5% as follows: a) in favor of TAP block, b) in favor of placebo, c) insignificant difference. 34 Second, the weighted outcome was estimated using the random effects method to determine the influence of between study heterogenity on the primary meta analytic estimation.

The quality of evidence and strength of recommendations of the results obtained in the present systematic review and meta-analysis were rated following the Grading of Recommendations Assessment, Development and Evaluation GRADE criteria. 35

## Results

### Study Selection

In our initial search, we identified 218 potentially relevant articles. Of those, 54 were identified in PubMed, 96 in Embase, 66 in Cochrane Library, 1 in LILACS, and 1 in gray literature. Of those, 31 were excluded after screening the title, 16 due to duplication, and thus 171 were selected for abstract reading. A total of 164 studies were excluded because they failed to meet the inclusion criteria. The remaining 7 studies 13 14 15 16 17 18 21 met the inclusion criteria and were included in our quantitative analysis, comprising 261 patients who underwent hysterectomy and were intervened with TAP block, who were compared with 257 patients who underwent hysterectomy and were intervened with sham block or who were not intervened. We documented the selection process with a Preferred Reporting Items for Systematic Reviews and Meta-Analyses

**Fig. 1:**
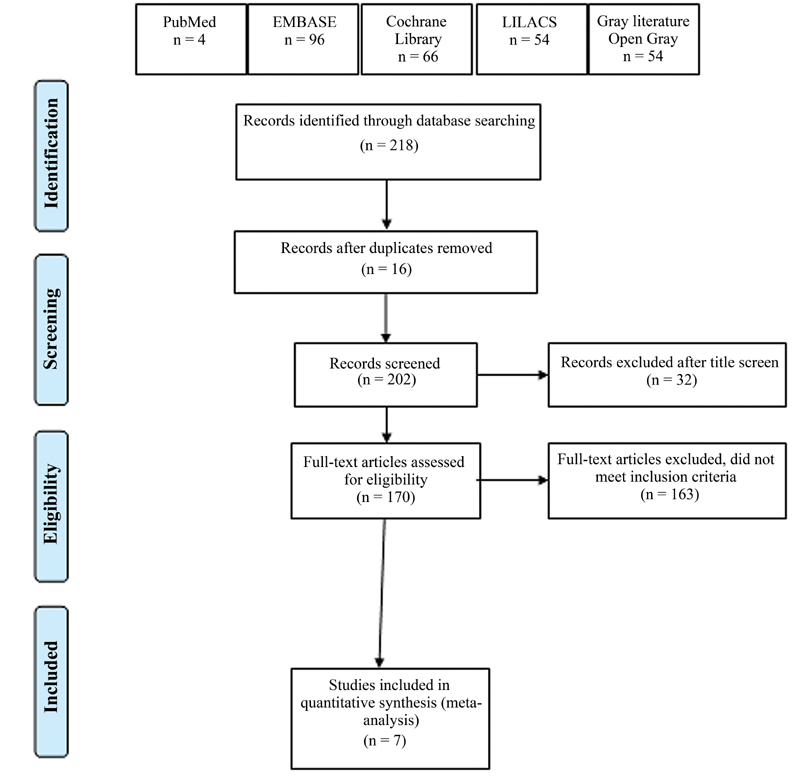
PRISMA flow diagram.

PRISMA flow chart ( Fig. 1 ).

### Study Characteristics

All of the analyzed studies were randomized control trials. Three of the studies 13 15 21 compared TAP block with sham block with saline, and the other four 14 16 17 18 with no treatment. In five of the studies, 13 14 15 16 18 TAP block was performed after laparoscopic hysterectomy; in addition the study by Kane et al. 14 from 2012 also included single-port hysterectomy, and the study by Bava et al. 17 from 2016 included laparoscopically-assisted vaginal hysterectomy, both of which are considered variants of laparoscopic hysterectomy and are all considered to be minimally invasive procedures. On the other hand, compared with the rest of the studies, which only included benign disease, the study by Torup et al. 21 from 2015 was performed exclusively in patients undergoing robot-assisted hysterectomy and included patients with malignant disease.

All of the analyzed studies reported postoperative pain scale scores as an outcome. Pain was assessed by the visual analogue scale (VAS) or by numeric rating scales. Six studies 13 14 15 16 18 21 assessed opioid requirement. The studies by Bava et al. 17 and Kane et al. 14 , from 2016 and 2012, respectively, additionally included among their outcomes the postoperative quality of recovery using the QoR-40 survey. Four studies 16 17 18 21 specifically assessed the incidence of postoperative nausea and vomiting as side effects related to the use of opioids. Sedation associated to the use of opioids was evaluated in the studies by Bava et al. 17 and Guardabassi et al. 18

The Ramsay sedation scale was used in five of the studies, which also reported the American Society for Anesthesiologists (ASA) classification, 13 15 16 18 21 and included patients classified as ASAI and ASAII, except in the studies by Ghisi et al. 16 and Torup et al. 21 , which also included patients classified as ASAIII. In five of the studies, 13 14 18 21 ropivacaine was the anesthetic used to perform TAP block, while bupivacaine was used in the study by Calle et al., 15 and Ghisi et al. 16 used levobupivacaine. In all of the analyzed studies, certified anesthesiologists performed the ultrasound-guided TAP block, except in the study by Calle et al., 15 in which surgeons performed laparoscopic-guided TAP block upon completion of surgical intervention ( Table 1 ).

**Table 1:** Characteristics of included studies evaluating the efficacy of TAP block after hysterectomy

Study	Country	Type of surgery	Inclusion criteria	Exclusion criteria	Outcomes	Comparison group	Number TAP / Control Sequence generation and concealment	Blinding	Anesthetic drug dose (mg)	ASA	TAP block technique
**Calle et al. (2014)** 15	Colombia Prado Clinic and CES University, Medellin	Laparoscopic	Patients with ASA surgical risk classification types 1 and 2; had no contraindications for administration of local anesthetics, NSAIDs, or acetaminophen; had an adequate level of understanding, i.e., being able to communicate by telephone and understand a numerical scale.	Change in the standard anesthetic technique, hospitalization following hysterectomy, previous medical history of allergy to local anesthetics, and not being able to be reached by telephone	Pain scale scores (VAS) at 24, 48, and 72 hours after surgery, opioid requirement after surgery	Placebo	100 / 97 Sequence was generated using computer-generated randomization list in blocks, which were placed in sealed envelopes	Triple blind: Patient, surgeon, and data analyst	Bupivacaine 0.25% (96)	I, II	Laparoscopic-guided
**De Oliveira et al. (2011)** 13	United States of America Northwestern University, Chicago	Laparoscopic	Healthy women undergoing laparoscopic hysterectomy	Patients with previous history of allergy to local anesthetics, long-term use of opioid analgesics or corticosteroids, and pregnancy	Quality of Recovery (QoR-40) at 24h; pain numeric scale score at 30 minutes, 60 minutes, and 24 hours; time to opioid requirement and cumulative opioid consumption at 24 hours, and number of postoperative antiemetics	Placebo	22 / 23 Individuals were randomized into three groups using a computer-generated table of random numbers, and group assignments were sealed in sequentially numbered envelopes	Double blind: patients, anesthesia care providers	Ropivacaine 0.5% 100	I, II	Ultrasound
**Ghisi et al. (2016)** 16	Italy Instituti Ospitalieri Cremona	Laparoscopic	Patients between 18 and 70 years old, undergoing elective total laparoscopic hysterectomy	Chronic opioid therapy in the previous 3 months before surgery, conversion to open surgical technique, BMI > 30 kg/m ^2^ or < 18 kg/m ^2^ , postoperative recovery in intensive care unit, chronic therapy with antidepressants, known diagnosis of epilepsy or therapy with antiepileptic drugs, bilirubin level > 3.0 mg/dL, aspartate aminotransferase and/or alanine aminotransferase > 250 IU, creatinine level >1.4 mg/dL, pregnancy or lactation, known allergy to any drug used in the study, local infection at the block site, and drug or alcohol addiction.	Postoperative pain at rest and during movement using NRS of 0 to 10, at 2, 4, 6, and 24 hours. Morphine requirement 24 hours, incidence of postoperative nausea and vomiting (PONV) using the Apfel score	No block	22 / 22 Patients were randomized into two groups using computer-generated sequence of numbers placed in sealed envelopes	Single blind: observer (data collection)	Levobupivacaine 0.375% (75)	I - III	Ultrasound
**Guardabassi et al. (2017)** 18	Argentina Hospital Italiano de Buenos Aires	Laparoscopic	Patients between 18 and 70 years old; BMI < 35 kg/m ^2^ ; undergoing total laparoscopic hysterectomy	Previous medical history of allergy to local anesthetics; psychiatric disorders or dementia, abdominal wall infection; chronic use of analgesics, chronic pain syndrome; diagnosed peripheral neuropathy; known allergy to analgesics or corticoids.	Pain NRS: non-randomized study scores at 60 minutes, 2, 8, and 24 hours after surgery; opioid consumption during the first 24 postoperative hours; adverse effects on quality of sleep of the first night after surgery; episodes of nausea and vomit; Ramsay sedation scale	No block	20 / 20 Non-probability sampling of consecutive case series. Random assignment using sealed envelopes	Single-blind: Data analysts	Ropivacaíne 0.5% (75)	I, II	Ultrasound
**Bava et al. (2016)** 17	China Department of Anesthesiology and Operation, Hospital of People's Liberation Army. Xi'an	Laparoscopic and LAVH	Women scheduled for elective laparoscopic hysterectomy with benign lesions	Patients with preoperative use of analgesics were excluded due to potential impact on postoperative analgesia requirement; BMI > 30 kg/m ^2^ ; coagulopathy; contraindication for peripheral nerve block; any drug allergy	Pain, with NRS: 30 and 60 minutes, 4, 8, 12, and 24 hours PONV, Ramsay sedation scale Satisfaction scores	No block	35 / 36 Computer-generated randomization list in blocks, placed in sealed envelopes	Double-blind: blinded to patients and data analysts, but not to members of the surgical and anesthesia care teams	Ropivacaine 0.375% (112.5)	—	Ultrasound
**Kane et al. (2012)** 14	United States of America Metrohealth Medical Center, Case Western Reserve University, Cleveland	Laparoscopic and single-port	All women undergoing laparoscopic hysterectomy by a single surgeon between April and September 2011 were approached to participate in this study.	Patients on chronic pain narcotic medications, or if they had allergy to local anesthetic.	Numeric visual analog scales for pain and opioid requirement at 2 and 24 hours after surgery; quality of recovery (QoR-40 survey) at postoperative day 1	No block	28 / 29 Computer-based block randomization	Single-blind: blinded to data analysts	Ropivacaine 0.5% with epinephrine (100)	—	Ultrasound

Abbreviations: ASA, American Society of Anesthesiology; BMI, body mass index; NRS, non-randomized study; NSAIDS, nonsteroidal anti-inflammatory drugs; PONV, Postoperative nausea and vomiting.

### Risk of Bias within Studies

In six of the studies, proper randomization procedure was followed, with random sequence generation and concealment, with balanced baseline characteristics between groups, thus suggesting no issues in the randomization procedure. In the remaining study, the randomization procedure was considered unclear, since nonprobability sampling of consecutive case series was used.

All of the analyzed studies were assessed as having low risk of deviation bias due to the planned intervention, since there was no evidence of cointerventions or changes in the treatment protocol, the interventions were successfully performed, and the participants were adherent to the assigned intervention. All of the studies were judged as having low risk of bias due to loss of outcome information, taking into account that results were available for most participants, that the studies exhibited proportions of missing data < 10%, and despite this their results were robust.

Six of the seven studies were judged as having low risk of measurement bias, since the evaluators were not aware of the performed intervention and, therefore, this could not influence their results. We considered the study by Kane et al. 14 to have an unclear risk of measurement bias, since the research coordinator or members of the surgical team, who were not blinded to treatment allocation, were the ones conducting the interviews at the hospital or by phone to apply the QoR-40 questionnaire.

All seven studies were considered to have a low risk of selection bias because the reported results were those asked in the goals, and measurements were made with previously validated scales. Furthermore, five of the seven studies were judged as having low risk of overall bias, since a biased direction toward the alternative hypothesis was not defined. On the other hand, the remaining two studies were considered as having unclear overall bias, since in the study by Guardabassi et al. 18 flaws in the randomization procedure were identified, and in the study by Kane et al. 14 the quality of recovery questionnaire was applied by members of the research team that were aware of the treatment allocation of the patients ( Table 2 ).

**Table 2:** Risk of bias domains and overall bias

Study (ref)	Randomization process	Deviation from planned intervention	Data on loss of results	Measurement of outcomes	Selection of reported outomes	Overall bias
Calle et al. (2014) 15	Low	Low	Low	Low	Low	Low
De Oliveira et al. (2011) 13	Low	Low	Low	Low	Low	Low
Ghisi et al. (2016) 16	Low	Low	Low	Low	Low	Low
Guardabassi et al. (2017) 18	Some concern	Low	Low	Low	Low	Some concern
Bava et al. (2016) 17	Low	Low	Low	Low	Low	Low
Torup et al. (2015) 21	Low	Low	Low	Low	Low	Low
Kane et al. (2012) 14	Low	Low	Low	Some concern	Low	Some concern

### Results of Individual Studies and Summary of Results

Study details, including demographic and operative characteristics are shown in Table 1 . In the study by Calle et al. 15 , the authors reported that patients receiving TAP block exhibited a statistically significant reduction in pain scale scores at time of hospital discharge compared with those in the placebo group ( *p* = 0.017). However, they concluded that the role of TAP block for this procedure was questionable because of the lack of clinical significance due to the small difference identified.

De Oliveira et al. 13 reported that cumulative opioid consumption during the first 24 hours after surgery was lower in the 0.5% ropivacaine group compared with saline ( *p* = 0.003). Linear regression showed an inverse relationship between opioid consumption and global quality of recovery at 24 hours in all three groups. Numerical pain rating scale scores in the recovery room were lower in the ropivacaine groups compared with saline. Thus, the authors concluded that preoperative TAP infiltration led to improved quality of recovery and analgesia in patients undergoing laparoscopic hysterectomy.

The study by Ghisi et al. 16 showed that morphine consumption was comparable between groups during their stay at the postanesthesia care unit and during the first 24 hours ( *p* = 0.154; *p* = 0.950). Numeric rating scale (NRS) scores for pain at awakening were also comparable between groups ( *p* = 0.086). This study concluded that ultrasound-guided TAP block did not reduce opioid consumption or pain scores at rest or movement during the first 24 hours after laparoscopic hysterectomy.

Similarly, the study by Guardabassi et al. 18 analyzed opioid consumption and scored pain using the visual numeric scale (VNS) during the first 24 postoperative hours, specifically at 60 minutes, 120 minutes, 8 hours, and 24 hours after surgery. The authors found no significant differences between groups in opioid consumption ( *p* = 0.2) and reported that differences in pain scale scores were not statistically significant ( *p* > 0.1) at the analyzed time points. Hence, they concluded that TAP block did not improve postoperative patient controlled opioid analgesia used for pain management in gynecologic laparoscopic surgery.

Bava et al. 17 reported that patients receiving TAP block exhibited a significantly lower NRS pain score compared with controls ( *p* < 0.05), as well as a reduced postoperative analgesic requirement. Satisfaction scores were significantly higher in the TAP block group (Z = 1.61; *p* < 0.01), and length of stay and adverse effects were comparable between groups ( *p* > 0.05). The authors concluded that, after laparoscopic hysterectomy, the joint use of TAP block and local anesthesia is a better analgesic approach compared with local anesthesia alone.

On the other hand, the study by Kane et al. 14 measured the early postoperative quality of recovery using validated QoR questionnaires and found no statistically significant improvement in scores of quality of recovery in the TAP block group (score 168; 125–195) compared with no block (score 169.5; 116–194) ( *p* = 0.533). Furthermore, no statistically significant difference was found between groups in narcotic use, which was 11.7 mg (0–24) of morphine in the TAP block group versus 11.8 mg (0–27) ( *p* = 0.474) in the no block group. Visual analog scale pain scores were also comparable between the TAP block (score 50.0; 0–100) and the no block group (score 60.0; 20–100) ( *p* = 0.447). In conclusion, no statistically significant differences were identified in pain scores, narcotic use, or quality of recovery in patients receiving TAP block after hysterectomy. Moreover, the authors highlight that there was a significant increase in the time required in the posthysterectomy operating room for the placement of the ultrasound-guided TAP block.

The study by Torup et al. 21 found no differences between groups in median morphine consumption during the first 24 hours after surgery, VAS scores, and frequency of postoperative nausea and vomiting. Thus, in this study, TAP block, in addition to a basic analgesia regime with paracetamol and NSAIDs, did not provide further reduction in morphine consumption, in VAS pain scores, or in the frequency of nausea or vomiting after robot-assisted hysterectomy.

### Early Postoperative Pain

This outcome was evaluated in all of the analyzed studies, 13 14 15 16 17 18 21 which altogether included a total of 518 patients. Our analysis showed that patients receiving TAP block reported statistically lower pain scale scores. Using the least squares method, the difference in means was of - 1.17 (95% confidence interval [CI]: - 1.87–- 0.46), with I ^2^ = 68% and Tau 2 = 2.65 (95%CI: 0.93–7.56), indicating intermediate heterogeneity.

### Late Postoperative Pain

**Fig. 2:**
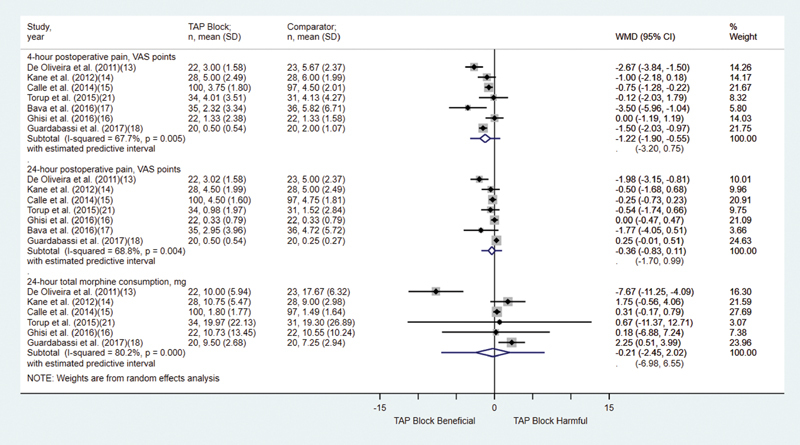
Forest Plot: 4h postoperative pain, 24h postoperative pain.

This outcome was evaluated in all of the analyzed studies, 13 14 15 16 17 18 21 which altogether included a total of 518 patients. Our analysis showed that there was no statistically significant difference in pain scale scores. Using the least squares method, the difference in pain scale scores between groups was not statistically significant between the 2 groups: 0.001 (95%CI: - 0.44–- 0.44), with I ^2^ = 69% and Tau 2 = 2.75 (95%CI: 0.96–7.84), indicating intermediate heterogeneity ( Fig. 2 ).

#### Opioid Requirement

**Fig. 3:**
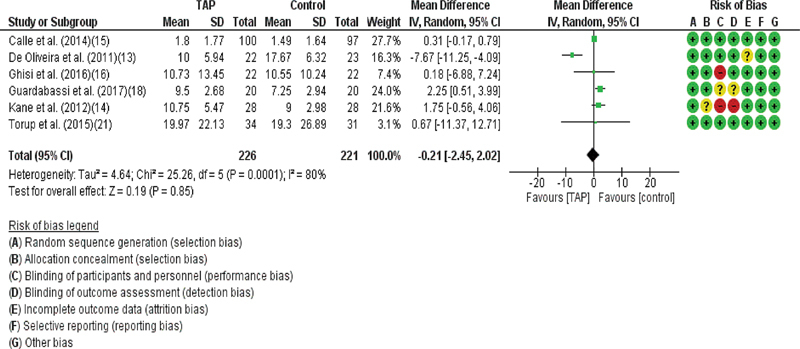
Forest Plot: total morphine consumption.

Six of the 7 studies evaluated opioid requirement. 13 14 15 16 18 21 Using the least squares method, the difference in opioid consumption was of 0.37 (95%CI: - 0.95–1.68), with I ^2^ = 80% and Tau 2 = 4.21 (95%CI: 1.36–13.05), indicating high heterogeneity ( Fig. 3 ).

### Nausea and Vomiting

Regarding presence of nausea and vomiting, our results indicate that this outcome showed a significant difference in favor of TAP block.

### Risk of bias across studies and sensitivity analysis

A high level of heterogeneity was identified through the use of fixed and random effect models of meta-analysis, and therefore we explored baseline characteristics 33 and performed a meta-analysis to assess whether the identified heterogeneity could be attributed to methodological flaws, which showed no statistically significant differences between intervention and control groups.

Patient age was the only characteristic reported by all of the studies, and the difference between groups was of 0.84 (95%CI: - 0.17–1.86) with I ^2^ = 17%. On the other hand, body mass index (BMI) was reported by 5 of the studies, and the difference of - 0.20 (95%CI: - 0.39–- 0.01; I ^2^ = 25%), was statistically significant. Surgical time was reported by 4 studies, and the difference was of - 3.77 (95%CI: - 8.44–0.90; I ^2^ = 0%).

**Fig. 4:**
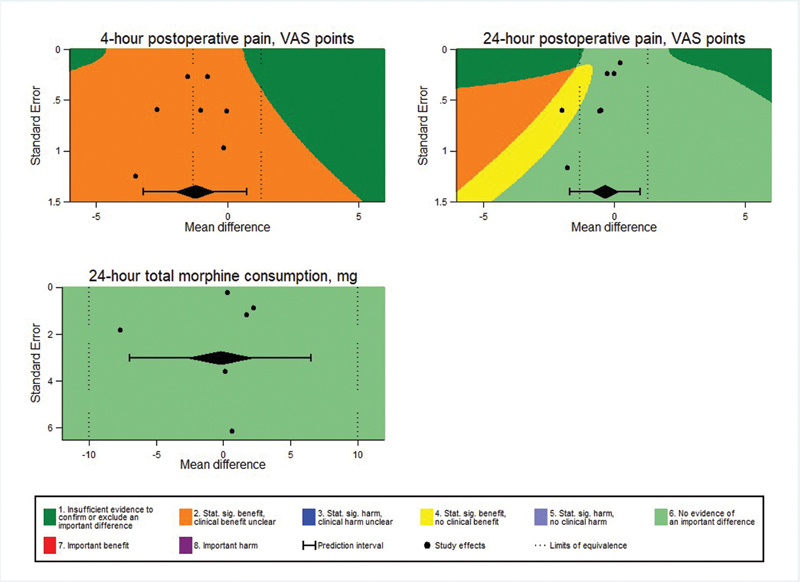
Forest Plots and Funnel plot augmentation.

Forest plots and funnel plot augmentations indicated robust results regarding precision and estimation of outcomes of TAP block regarding reduction of late postoperative pain and opioid requirement. The prediction interval showed that, given the current data, it is unlikely that new studies show opposite results. On the other hand, it is not clear whether the inclusion of new studies would change the results regarding the efficacy of TAP block in reducing early postoperative pain ( Fig. 4 ).

## Discussion

### Summary of Evidence

Over the past decade, minimally invasive surgery has gained relevance because of its advantages, including its association with less postoperative pain. 36 However, patients undergoing laparoscopic hysterectomy reported considerable pain of multifactorial origin, including somatic, visceral, and referred. 8 Consequently, these patients may require large amounts of opioid during the first 24 hours after surgery. 37

Transversus abdominis plane block has been the matter of study in multiple trials, whose results have allowed its use in open and laparoscopic gynecological surgery. However, the results of studies reporting the efficacy of TAP block regarding reduction of pain and opioid requirement are contradictory, generating confusion in deciding whether to use it in clinical practice and to include it as part of multimodal analgesia protocols.

In the present meta-analysis, our results suggest that TAP block improves early postoperative pain scale scores, with a statistically significant difference of - 1.17 (95%CI: - 1.87–- 0.46), taking into account that a decrease of 1 point in pain scores, or of 15 to 20%, is considered as a minimum change, and to generate clinically significant improvement for patients, such as the lack of need to request rescue medication, pain scale scores should be reduced by 2 to 2.7 points, or by 30 to 40%. 27 28 29 30 Therefore, the clinical relevance of this finding is questionable, and the clinical benefit is unclear according to the prediction interval result.

Results of this outcome are similar to those found in other meta-analyses. 22 23 Differences in postoperative pain reduction did not go beyond the first 4 hours, and this difference was not significant 24 hours after surgery: 0.001 (95%CI: - 0.44–0.44), which is in agreement with the findings of the meta-analysis by Tubog et al. 22

The type and dose of medications used in the different studies were equivalent; however, to explain the high level of heterogeneity identified, we conducted a sensitivity analysis excluding studies using bupivacaine 15 and levobupivacaine, 16 which showed the same results for the analyzed outcomes: early VAS score: - 1.65 (95%CI: - 2.47–- 0.83; Tau: 0.44; I ^2^ : 55%); late VAS score: - 0.74 (95%CI: - 1.71–0.23; Tau: 0.85; I ^2^ : 78%); and opioid requirement: - 0.80 (95%CI: - 5.33–3.74; Tau: 16.07; I ^2^ : 88%).

Regarding presence of nausea and vomiting, our results indicate that this outcome showed a significant difference in favor of TAP block. In the meta-analysis by Bacal et al., 23 due to the lack of consistency between studies, they were unable to evaluate the role of TAP block in the incidence of postoperative nausea and vomiting.

One of the strengths of the present study is the emphasis placed on our analysis to explain the high heterogeneity found between studies, and while it is not possible to conclude conclusively, we consider that the simple randomization process may play an important role in the persistence of such a high heterogeneity and affect the observed causal inference, which was taken into account when concluding about our findings.

Additional strengths include the exclusive use of clinical trials with systematic and methodological application of inclusion and exclusion criteria, whose quality was assessed by evaluation of risk of bias, which was low in general for all studies, and intermediate for two of the studies in relation to randomization in the study by Guardabassi et al. 18 and to outcome measurement in the study by Kane et al. 14 . However, this did not affect the reported results.

Among the limitations identified are the use of no block in four of the studies, which may lead to results that are less robust compared with placebo. Nonetheless, the results were similar for these two types of comparators. No particular study explains the obtained I ^2^ value, and the present meta-analysis was unable to explain the high heterogeneity between studies.

The comparability between studies may be affected by factors such as the use of different protocols regarding who did the intervention and measured outcomes, different blinding techniques, the report of a previous explanation to patients of the pain scales used, ambulatory management for some, and the intra- and postoperative analgesia regime used.

The analyzed studies did not report on comorbidities that may influence the intensity and duration of postoperative pain, such as endometriosis and chronic pelvic pain. Unfortunately, the only baseline characteristic reported by all studies was patient age, making the comparison of groups between studies challenging, as did the inconsistency in reporting adverse effects of opioid use; thus this should be taken into account in future studies.

Moreover, some of the studies did not perform an intent-to-treat analysis, which may affect the results. To obtain unreported measures, we contacted the corresponding author of three of the studies, of which only one replied. Therefore, the use of spreadsheets developed for graphs with unavailable numerical data was required.

## Conclusion

In conclusion, the results obtained in the present meta-analysis indicate that TAP block improves early postoperative pain as indicated by the statistically significant difference; however, the clinical benefit of this difference is unclear. We did not find relevant evidence to suggest that there were any significant differences in late postoperative pain and in opioid requirement in patients receiving TAP block. Based on the best available evidence to date, we conclude that TAP block should not be considered as an effective analgesic technique to improve postoperative pain in patients undergoing laparoscopic or robotic hysterectomy. This was concluded based on the obtained results, which showed a marginal analgesic efficacy in the early postoperative period albeit of unclear clinical relevance, and that this effect was not maintained over time nor decreased the opioid requirement. Nonetheless, since the evidence synthesis showed high heterogeneity and the baseline characteristics exhibited imbalance, the strength of the evidence resulting from the present meta-analysis is rated as moderate, as determined by the Grading of Recommendations Assessment, Development and Evaluation (GRADE) criteria, despite being randomized controlled clinical trials. On the other hand, regarding the small sample sizes in these trials, it is important that future studies take into consideration the use of efficient randomization methods in regard to balancing baseline characteristics. In addition, preoperative conditions that may modify outcomes, such as diseases associated with pelvic pain, should also be taken into account to generate stronger evidence.
